# Changes in Cancer Cell Metabolism Revealed by Direct Sample Analysis with MALDI Mass Spectrometry

**DOI:** 10.1371/journal.pone.0061379

**Published:** 2013-04-26

**Authors:** David A. Pirman, Ekem Efuet, Xiao-Ping Ding, Yong Pan, Lin Tan, Susan M. Fischer, Raymond N. DuBois, Peiying Yang

**Affiliations:** 1 Department of Cancer Biology, The University of Texas MD Anderson Cancer Center, Houston, Texas, United States of America; 2 Department of General Oncology, The University of Texas MD Anderson Cancer Center, Houston, Texas, United States of America; 3 Department of Molecular Carcinogenesis, The University of Texas MD Anderson Cancer Center, Houston, Texas, United States of America; University of South Alabama, United States of America

## Abstract

Biomarker discovery using mass spectrometry (MS) has recently seen a significant increase in applications, mainly driven by the rapidly advancing field of metabolomics. Instrumental and data handling advancements have allowed for untargeted metabolite analyses which simultaneously interrogate multiple biochemical pathways to elucidate disease phenotypes and therapeutic mechanisms. Although most MS-based metabolomic approaches are coupled with liquid chromatography, a few recently published studies used matrix-assisted laser desorption (MALDI), allowing for rapid and direct sample analysis with minimal sample preparation. We and others have reported that prostaglandin E_3_ (PGE_3_), derived from COX-2 metabolism of the omega-3 fatty acid eicosapentaenoic acid (EPA), inhibited the proliferation of human lung, colon and pancreatic cancer cells. However, how PGE_3_ metabolism is regulated in cancer cells, particularly human non-small cell lung cancer (NSCLC) cells, is not fully understood. Here, we successfully used MALDI to identify differences in lipid metabolism between two human non-small-cell lung cancer (NSCLC) cell lines, A549 and H596, which could contribute to their differential response to EPA treatment. Analysis by MALDI-MS showed that the level of EPA incorporated into phospholipids in H596 cells was 4-fold higher than A549 cells. Intriguingly, H596 cells produced much less PGE_3_ than A549 cells even though the expression of COX-2 was similar in these two cell lines. This appears to be due to the relatively lower expression of cytosolic phospholipase A_2_ (cPLA_2_) in H596 cells than that of A549 cells. Additionally, the MALDI-MS approach was successfully used on tumor tissue extracts from a K-ras transgenic mouse model of lung cancer to enhance our understanding of the mechanism of action of EPA in the *in vivo* model. These results highlight the utility of combining a metabolomics workflow with MALDI-MS to identify the biomarkers that may regulate the metabolism of omega-3 fatty acids and ultimately affect their therapeutic potentials.

## Introduction

While much effort has been devoted to genomic profiling leading to the identification of certain gene components of cancer, information on metabolomics and lipidomics in cancer cells or tissues is limited. Even though metabolomics and lipidomics pose technological challenges in terms of instrument capability, reproducibility, and data handling, these fields of study show great promise in providing a comprehensive overview of a cancer cell's metabolism, thus leading to new and potentially personalized therapies.

Recent advancements in matrix-assisted laser desorption/ionization (MALDI) mass spectrometry (MS), [1]–[7] have increased MALDI's utility across a broad range of new applications. Advancements in commercially available instrumentation, including MALDI coupled to the linear ion trap Orbitrap instrument or quadrupole-time-of-flight (QTOF) mass spectrometers have allowed for the successful generation of mass spectra in the lower mass ranges (<1000 Daltons), thus allowing for MALDI-MS profiling of metabolites including lipids, not typically common with MALDI instrumentation due to matrix effects and source fragmentation [7]–[9]. These advancements in MS and ionization instrumentation have moved MALDI-MS into numerous new research areas, including lipidomics [7], [10]–[12], peptide [2], drug [13]–[16], and metabolite [17] analyses directly from biological samples, including tissues.

MALDI-MS has recently been used successfully to directly analyze biological samples combined with statistical data handling. These approaches can be used to differentiate tissue types or identify differentially regulated metabolism pathways. For example, the intra-surgical probes being developed by Balgo, *et al*., in which tissue is sampled during surgical resection, analyzed by MS, can then be categorized with statistical software [18]. This approach has allowed for rapid, unbiased discrimination of diseased and healthy tissue and could potentially be used to replace traditional histological classification during surgical resection of a tumor. Recently, MALDI-MS has also been used to classify tumor grades as well as tumor origin, although not intrasurgically [5], [19]–[21]. These recently published studies highlight the expanded use of MALDI-MS for direct tissue analysis to characterize tissues on the basis of their metabolite, lipid, peptide, and protein profiles.

In the current study, we used MALDI coupled to a QTOF mass spectrometer for the rapid interrogation of two NSCLC cell lines to reveal differences in the cellular metabolism of the fish-oil-derived, poly-unsaturated fatty acid (PUFA) eicosapentaenoic acid (EPA). We also adapted the technique to directly analyze and characterize tissue lipid metabolism of EPA from EPA-treated K-ras transgenic mouse lung tumors by both liquid chromatography coupled with tandem mass spectrometry (LC-MS/MS) and MALDI-MS to verify the *in vitro* MALDI data using a complementary approach.

**Figure 1:**
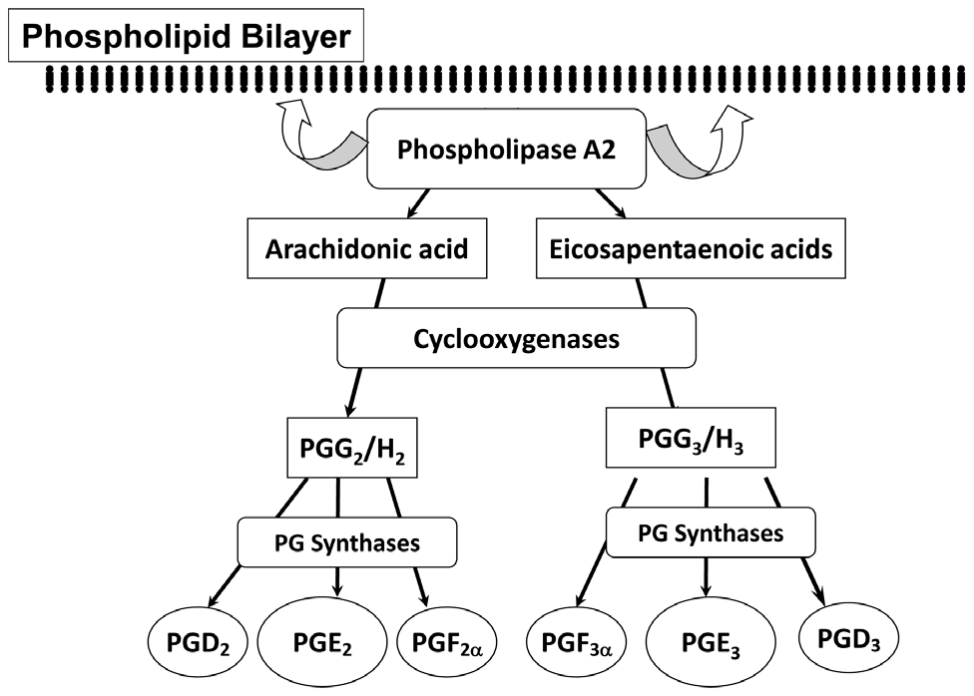
Biosynthesis of prostaglandin (PG) 2 series and PG3 series by AA or EPA through cyclooxygenase (COX) enzymes.

Numerous preclinical studies have supported the notion that the fish-oil-derived omega-3 fatty acids EPA and docosahexaenoic acid (DHA) have the ability to prevent cancer cell proliferation, migration, and invasion in various tumor types, including NSCLC [22]. We and other investigators have reported that the effectiveness of EPA could be mediated through its cyclooxygenase (COX) metabolite prostaglandin E_3_ (PGE_3_) in human lung, colon, and pancreatic cancer cells [23]–[25]. In contrast to DHA, EPA can also act as a substrate of COX, particularly COX-2, leading to an increase in PGE_3_ as opposed to arachidonic acid (AA) which gives rise to the pro-inflammatory metabolite prostaglandin E_2_ (PGE_2_) (Fig. 1) [22]. High levels of COX-2 activity, and thus PGE_2_, are known to be associated with increased cell proliferation and metastatic potential in tumors [26]–[28]. EPA has previously been shown to be effective in reducing A549 cell proliferation by increasing the production of PGE_3_ and thus, increasing the PGE_3_/PGE_2_ ratio [22]. However, how other factors associated with prostaglandin synthesis affect the anti-cancer effect of EPA in NSCLC cells has not been fully evaluated.

Herein, using MALDI-MS on two NSCLC cell lines that express similar levels of COX-2, we successfully identify differences in the cellular metabolism of EPA. With this technique, we were able to directly and rapidly (analysis time <30 seconds) interrogate the differential metabolism, particularly lipid metabolism, in each cell line in a reproducible manner. Different EPA-derived lipid metabolism was also observed in lung tumor tissues derived from the *K-ras* mutant mouse. Previous studies have shown successful protein profiling of human lung cancer subtypes using MALDI-MS [29], but here we showed the potential of MALDI for not only discriminating cancer subtypes by their distinct lipid profiles but also for tracking biological efficacy of treatment through the identification of adequate biomarkers. To our knowledge, this is the first report to directly analyze cancer cells by MALDI-MS, revealing differences in cellular metabolism, which could be critical for EPA-elicited anticancer activities in NSCLC cells.

## Methods

### Cell lines

The human non-small cell lung cancer (NSCLC) A549 and H596 cells were obtained from the American Type Culture Collection (Manassas, VA, USA) and maintained in a humidified atmosphere containing 5% CO_2_ at 37°C. A549 and H596 cells were routinely cultured in DMEM/F12 media (Invitrogen, Grand Island, NY, USA) supplemented with 5% and 10% heat-inactivated fetal bovine serum (Hyclone Laboratories, Logan, UT, USA), respectively, and Penicillin-Streptomycin 100× Solution and 2 mM L-glutamine from GIBCO (Invitrogen).

### Immunoblotting

For Western blot, cells at 70% confluency were washed twice with phosphate-buffered saline (PBS), trypsinized and cell pellets were collected. The pellet was washed twice with cold PBS and the resulting pellet was resuspended in lysis buffer (Invitrogen), sonicated immediately or stored at −80°C. Cell lysates were sonicated on ice for 3 min (Misonix Sonicator 3000, Farmingdale, NY, USA), and centrifuged at 14,000 rpm for 15 min at 4°C. Protein levels were quantified via the BioRad Dc protein assay (BioRad, Inc., Hercules, CA). Equal levels of protein (50 µg) were resolved on 7% (cPLA_2_) and 10% (COX-2) SDS PAGE gels and then transferred onto polyvinylidene difluoride membranes, according to standard methods. Following a 2-hr incubation in 5% nonfat dry milk blocking buffer prepared in Tris-buffered saline with 0.1% Tween 20 (TBST), membranes were probed with cPLA_2_ primary antibody (Santa Cruz Biotechnology, Santa Cruz, CA, USA) or the COX-2 (Cayman Chemical, Ann Arbor, MI, USA) at 1∶1000 dilution for 2 hrs, washed in TBST, incubated in secondary antibodies for 1 hr, followed by 3×10 min wash each in TBST. Protein bands were visualized via chemiluminesence using the ECL+ detection kit and hyper-film (Amersham Biosciences, Piscataway, NJ, USA). Equal loading of samples was illustrated by Western blotting for the presence of β-actin.

### cPLA_2_ activity assay

The activity of cPLA_2_ was determined using a cPLA_2_ assay kit (Cayman Chemical Company). Briefly, A549 and H596 (5×10^6^) cells were seeded in 100 mm dishes and allowed to grow overnight to reach ∼70% confluency. Cells were washed twice with cold buffer (50 mM Hepes, pH 7.4; 1 mM EDTA), scraped with a cell lifter (Corning Incorporated, Corning, NY, USA) and transferred to an eppendorf tube on ice. Following sonication for 3 min, the lysates were centrifuged at 14,000 rpm for 15 min at 4°C. The supernatant was removed and the concentration of proteins was determined by the BioRad Dc protein assay. For the activity assay, the volume equivalent of 1 µg of protein was used. The absorbance was read at 414 nm on a Spectra Max M5 plate reader (Molecular Devices Corp., Sunnyville, CA, USA). The cPLA_2_ activity was expressed in µmol/min/ml as determined by the formula provided in the manufacturer's protocol.

### K-ras transgenic mouse

All animal experiments were approved by the Institutional Animal Care and Use Committee at The University of Texas MD Anderson Cancer Center. To assess the lipid metabolism of EPA in mouse lung tissues, five-week-old *K-rasLA*C57Bl6/129/sv F1 mutant mice were used as a spontaneous lung tumor model and fed a diet containing soybean oil or EPA (1% and 2%) for 9 weeks. At the end of the ninth week, the mice were sacrificed, the lung tissues were removed, flash-frozen in liquid nitrogen and stored at −80°C until further analysis by MALDI-MS.

### Analysis by MALDI-MS

Cells (1×10^6^) were plated in 6-well plates and grown overnight. Cells were then treated with EPA (25, 50, or 100 µM), in serum-free media containing 1% BSA for 24 hrs. At the end of the incubation period, intact cells were collected by trypsinization, centrifuged at 3000 rpm for 2 min at 4°C, washed in PBS, and the cell pellet was resuspended in 20 µL of PBS. The cells were either stored at −80°C or immediately processed for MALDI-MS analysis.

For MALDI-MS analysis of cells, cell pellets were thawed and 1 µL of the suspension was spotted onto a polylysine-coated glass slide (Fisher Scientific, Pittsburgh, PA, USA) used as a MALDI target. For MALDI-MS analyses of tissue samples, 10−30 mg of tissue was homogenized in 500 µL of PBS using a Precellys tissue homogenizer (Bertin Technologies, Paris, France). An aliquot of 1 µL of the homogenate was spotted onto a polylysine-coated glass slide. Cell suspensions and tissue homogenate were dried in a vacuum desiccator at room temperature for 10 min. Dihydroxybenzoic acid (DHB) (20 mg/ml) in chloroform/ethanol (9∶1; *v/v*) was applied to the samples by spray-coating through a Meinhard nebulizer (Meinhard, Golden, CO, USA). The chloroform/ethanol mixture allowed rapid crystal formation over the cells while preventing pooling of the matrix solvent. Mass spectra were collected on a Waters Synapt G1 QTOF mass spectrometer (Milford, MA, USA). For lipid identification, accurate mass and MALDI-MS/MS data were collected on a Thermo Scientific MALDI LTQ Orbitrap mass spectrometer (San Jose, CA, USA).

### LC-MS/MS

Prostaglandins E_2_ and E_3_ (PGE_2_ and PGE_3_), were extracted according to the previously published method from Yang *et al*. [30], [31]. Briefly, an aliquot of the 0.5 mL of PBS buffer was added to the frozen tissue or cell pellets, followed by homogenization in 1.5 mL tubes with ceramic beads using the Precellys tissue homogenizer (Bertin Technologies) and 400 µL aliquots were used for prostaglandin extraction. All extraction procedures were performed under minimal light. Samples were then reconstituted in 100 µL of methanol/0.1% acetic acid (50∶50, *v/v*) prior to analysis by LC-MS/MS [31].

The extracellular levels of PGE_2_ and PGE_3_ in A549 and H596 cells were extracted according to Yang *et al* [22]. PGE_2_ and PGE_3_ were quantified by LC-MS/MS using an Agilent 6460 triple quadrupole (QqQ) mass spectrometer (Agilent Technologies, Palo Alto, CA, USA) equipped with an Agilent HP 1200 binary pump HPLC inlet (Agilent). PGE_2_ and PGE_3_ were separated using a 2×100 mm Kinetex 3 μ C18 analytical column (Phenomenex, Torrance, CA, USA). The mobile phase consisted of 0.1% formic acid and acetonitrile with 0.1% formic acid. The column temperature was maintained at 40°C, and samples were kept at 4°C during the analysis. Individual analytes were detected using electrospray ionization and multiple reaction monitoring, and the following *m/z* transitions were monitored in negative ionization mode with: *m/z* 351 →271 for PGE_2_, *m/z* 349 → 269 for PGE_3_, and *m/z* 355 → 275 for PGE_2_-d_4_. The levels of PGE_2_ and PGE_3_ were quantified using authentic standard curves and normalized to either the number of cells (intracellular or extracellular) or the amount of protein determined by a Bradford assay (Bio-Rad).

### Statistical analysis

Each biological replicate was spotted twice and analyzed twice by MALDI-MS and the experiments were repeated three times. Exported spectra from each group were uploaded into the Metaboanalyst online software (Metaboanalysis 2.0, Available: http://www.metaboanalyst.ca/. Accessed May 2012) [32], [33]. This online software offers an array of statistical tools to reduce the complex spectra and identify significantly changing *m/z* values. Principal component analysis (PCA), partial least-square discriminate analysis, t-tests, and analysis of variance were used to analyze the resulting MS data both to identify changing peaks and to determine whether the cell types or tissues could be discriminated from each other. Significantly different features were then compared with those from both the Human Metabolome Databank (HMDB; Available: http://www.hmdb.ca. Accessed May 2012) and the METLIN (Available: http://metlin.scripps.edu/. Accessed May 2012) database for compound characterization.

## Results

### EPA metabolism was differentially regulated in NSCLC A549 and H596 cells

**Figure 2:**
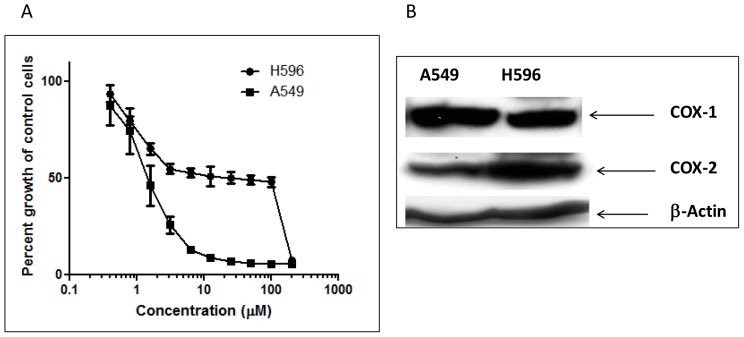
Western-blot showing similar COX-2 levels between both A549 and H596 (**A**). The anti-proliferative effect of EPA in human non-small-cell lung cancer A549 and H596 cells (B). Exposure of A549 cells to EPA for 72 hrs produced a ten-fold stronger inhibition of cell proliferation in A549 cells than that in H596 cells.

**Figure 3:**
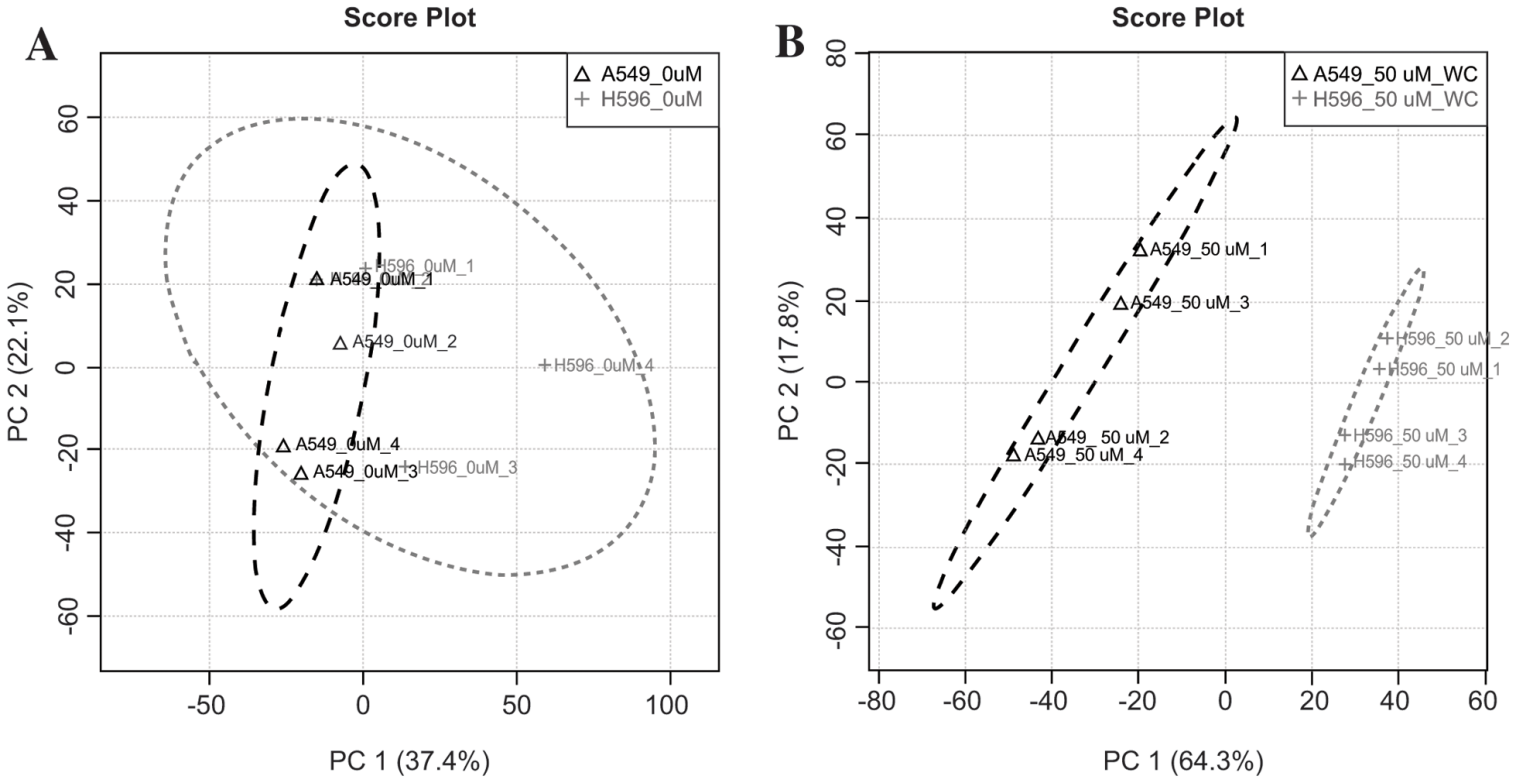
Representative PCA score plots of mass spectra collected directly from dried A549 and H596 cell spot by MALDI-MS. Cells were untreated (A) and treated with 50 µM EPA (B). Untreated A549 and H596 cells had similar mass spectra (A). However, post-EPA treatment led to a clearly differentiated metabolic pattern between these two cell lines (B). The data are representative of two biological replicates with repeated analysis. An average of 25 mass spectra were collected and averaged from each cell spot. The amount of time for each analysis was less than one minute. The data were represented from three replicated experiments.

**Figure 4:**
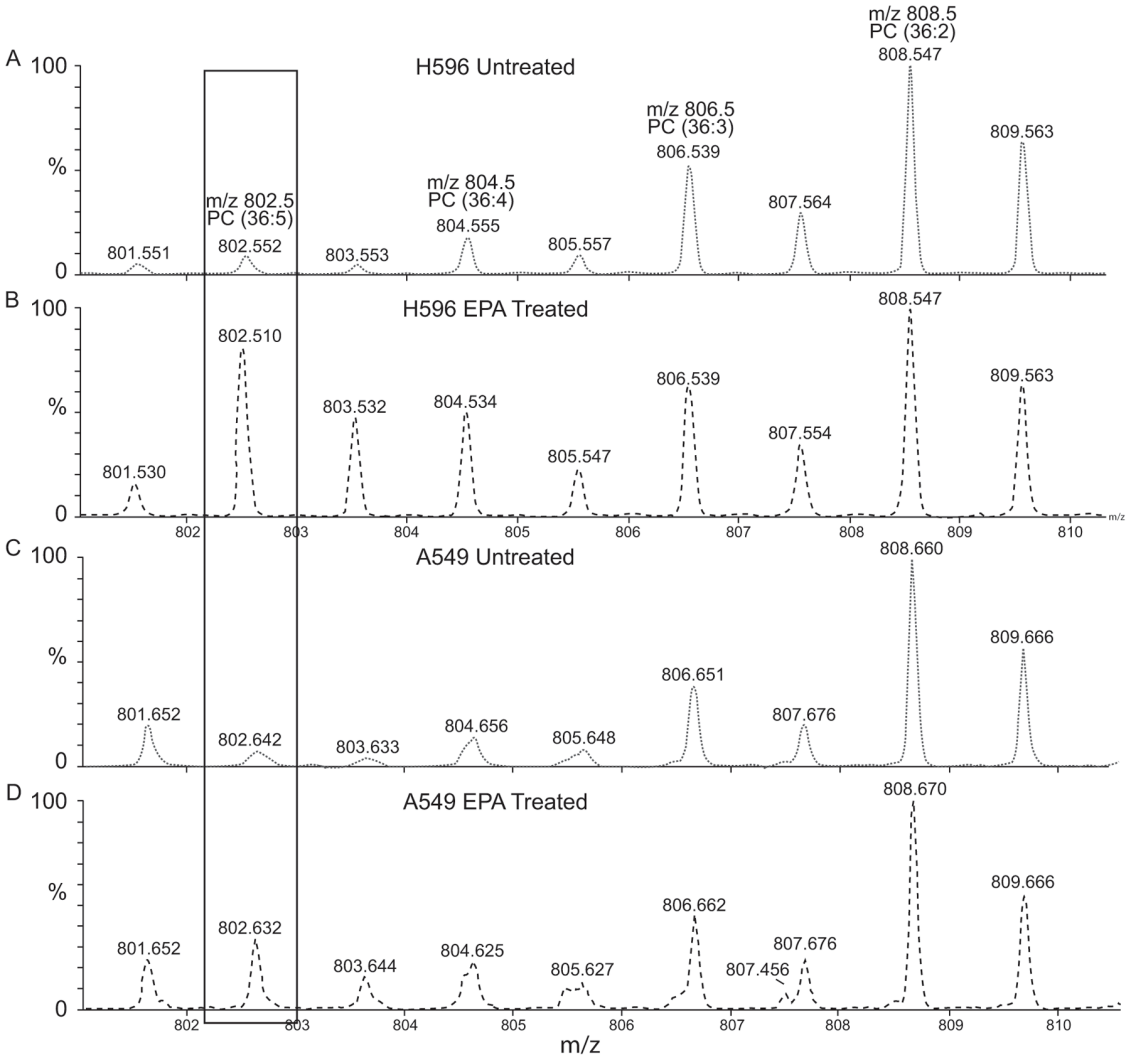
Representative mass spectrum comparing the spectral region from *m/z* 800−810 from H596 untreated (A) and EPA treated (B) cells. A significant increase is observed for *m/z* 802.5, which corresponds to a PC (36∶5) fatty acid. MS/MS confirmed that PC (16∶0/20∶5) was a component of the observed *m/z* value (data not shown). Spectra shown in (C) and (D), corresponding to the A549 cells line untreated and EPA treated; respectively, also show an increase in *m/z* 802.5 after treatment with EPA. However, this increase is significantly less than the increase observed for the H596 cell line.

As we reported previously, the anti-proliferative effect of EPA in human NSCLC cells were mediated through their expression of COX-2 and formation of the anti-proliferative metabolite, PGE_3_ [22]. However, when we treated two NSCLC A549 and H596 cells with EPA, EPA inhibited the proliferation of A549 cells (IC_50_<6.25 µM) more effectively than it did the H596 cells (IC_50_>50 µM) (Fig. 2A) even though these two cell lines showed similar COX-2 expression (Fig. 2B). To delineate the mechanisms that would mediate the differential effects noted with EPA treatment, we evaluated the different mass spectral profiles of these cell lines using MALDI-MS followed by PCA analysis (Fig. 3). Initially this analysis was simply used to determine if the collected MS spectra could indeed be used to discriminate between cell type and treated versus untreated samples. Significantly changing peaks identified by p-values <0.05 were investigated and identification attempted including the PC lipids further discussed. The MALDI-MS spectra of untreated A549 and H596 cells showed very similar metabolic/lipidomic signatures (Fig. S1A and S1B) aside from a few minor variations in signal intensities and thus not differentiated by PCA analysis, EPA-treated A549 and H596 cells differed substantially in their metabolic/lipidomic profiles (Fig. 4). After EPA treatment, the metabolite pattern in the two cell lines were easily differentiated by observing novel and up-regulated peaks, suggesting that cellular metabolism of EPA in these two cell lines is differentially regulated (Fig. S1C and S1D).

### Higher level of EPA incorporation into phosphatidylcholine (PC) in H596 than in A549 cells

Further inspection of the collected spectra offers insight into the differentially regulated EPA metabolism in these two particular cell lines, shown in Fig. 4. The peaks displayed in this figure represent lipid species with both varying acyl-chain lengths and degrees of unsaturation. For instance, *m/z* 804.5 represents the sodiated PC containing two acyl-chains totaling 36 carbons with 4 double bonds (36∶4). From previously published results, a reasonable assumption can be made that one acyl-chain contains 16 carbons with zero double bonds and that the other chain contains 20 carbons and four double bonds (an AA moiety) [7]. Most commonly, the unsaturated moiety occupies the SN2 position on the phosphoglycerol backbone; however, as lipid degradation and regeneration is continually in flux, the structures are continuously changing, making absolute lipid structural determinations difficult at any given time. Fig. 4B also shows a five-fold increase in *m/z* 802.5, [M+Na]^+^ of PC in H596 cells after treatment with EPA compared to that of cells treated with vehicle alone. Further MALDI-MS/MS analysis identified this mass to indeed consist of a PC most likely derived from EPA as PC (36∶5) (Fig. S2). The observed increase of PC (36∶5) is attributed to the incorporation of the EPA fatty acid at the PC, possibly at the SN2 position. In comparison, the similar metabolite from the A549 cells (Fig. 4C and 4D) shows only a marginal increase in *m/z* 802.5. Therefore, these differences in cellular metabolism can specifically be correlated to H596 cells upon EPA treatment. Comparison of the untreated cell spectra of the PC (36∶4) intensities in both cell lines resulted in similar values, indicating these two cell lines have similar ability to form such PC species. This result suggests that A549 and H596 cells differ minimally in the biosynthesis of PC but could have different abilities to regulate the utilization of EPA from PC.

To generate comparable estimations between the two cell lines and to account for any MALDI signal intensity variations, each fatty acid PC (*m/z* 802 and 804) was normalized to the signal intensity of the PC head group (*m/z* 184), which mainly forms via source fragmentation. Assuming the total PC content between the two cell lines is similar, this ion serves as a suitable normalizing ion, as it can be used to represent the entire PC species. This approach was limited to PC species with a 16∶0 acyl-chain at the SN1 position and the PUFA substitution at the SN2 position for simplicity of comparison. Similar calculations could be made with increasing SN1 acyl-chain length. From the calculations, a significant increase in PC (36∶5) and PC (36∶4) is observed in H596 cells after treatment with EPA (Table 1). The increase in PC (36∶5) can be attributed to the formation of PC species with an EPA moiety added at the SN2 position of the PC groups. The increase in the PC (36∶4) is indicative of the shift away from AA entering the COX-2 pathway towards that of EPA. An increase in both PC species is also observed in the A549 cell line; however, this effect is not as profound as that observed in the H596 cell line.

**Table 1:** Ratio of PUFA PC to PC head group.

	PC (36∶4): AA *m/z* 804/184	PC (36∶5): EPA *m/z* 802/184
**Untreated H596**	8.2±1.5%	0.1±0.1%
**EPA Treated H596**	25±0.8%	51±1.6%
**Untreated A549**	2.4±1.0%	0%
**EPA Treated A549**	4.2±0.1%	12±1.0%

Note: Mass spectral intensity data was normalized to the PC head group intensity (*m/z* 184) observed in the spectra resulting from source fragmentation. This can be used to represent the entire PC species in the biological sample.

Abbreviations: PUFA, poly-unsaturated fatty acids; PC, phosphitydalcholine lipid; AA, arachidonic acid; EPA, eicosapentaenoic acid; *m/z*, mass-to-charge ratio.

### H596 generated lower levels of PGE_3_ than did A549 cells

Since the MALDI-MS data suggest that EPA was primarily observed in a phospholipid form in H596 cells but not in A549 cells and both of these cells have similar phospholipid biosynthetic capabilities, our current hypothesis is that there might be differences in either the expression or activity of phospholipase A_2_ (cPLA_2_), an enzyme that releases fatty acids from the SN2 position of the glycerol backbone. This alteration could lead to the differing abilities of H596 and A549 cells to form PGE_3_ upon substrate availability, thus limiting the anti-proliferative effects of EPA in the H596 cells.

**Figure 5:**
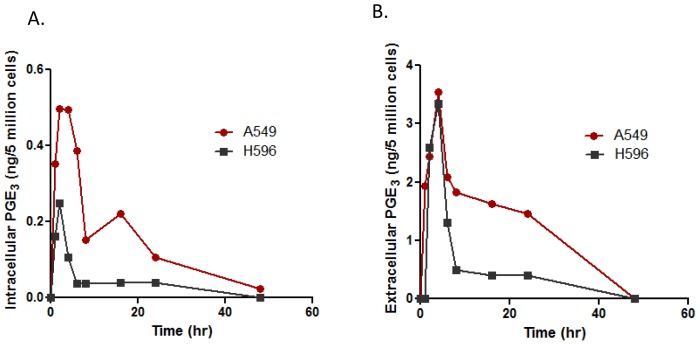
Intracellular and extracellular PGE_3_ in A549 and H596 cells. (A) Cells were treated with EPA for different times as indicated and intact cells were collected by trypsinization and subjected to analysis by LC-MS/MS. Intracellular levels of PGE_3_ in A549 cells were at least twofold higher than those in H596 cells. (B) Cell culture media were collected at different times as indicated and subjected to solid-phase extraction. Extracellular levels of PGE_3_ were then analyzed by LC-MS/MS. Data are representative of two separated experiments.

**Figure 6:**
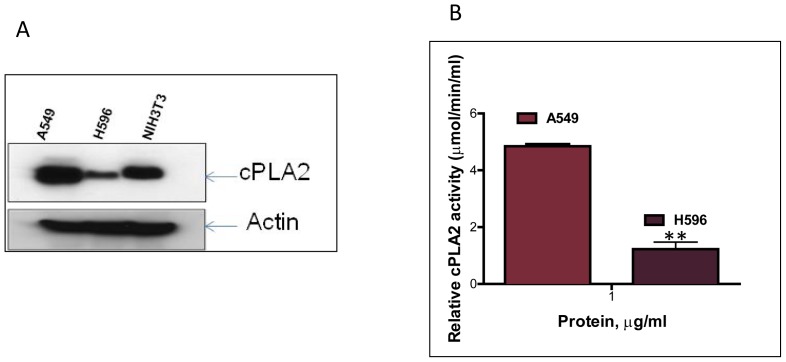
cPLA2 was differentially regulated in A549 and H596 cells. (A) Protein expression of cPLA2 in A549 and H596 cell was determined by Western blotting; (B) Activity of cPLA2 in A549 and H596 cells was measured as previously described. Both protein level and activity of cPLA2 were significantly lower in H596 cells compared to that of A549 cells.

We first compared the intracellular and extracellular levels of PGE_3_ in H596 and A549 cells treated with EPA (50 µM). As shown in Fig. 5, the intracellular level of PGE_3_ was almost two- to four-fold higher, at the time points up to 20 hrs in A549 cells than in the H596 cells, while a higher amount of extracellular PGE_3_ was observed in A549 cells than in H596 cells after 4 hrs of treatment. To further test our hypothesis, we determined the protein level of cPLA_2_ as well as its activity in these cells. As shown in Fig 6., the protein expression of cPLA_2_ was notably higher in A549 cells than that of H596 cells (Fig. 6A). Similarly, the activity of cPLA_2_ was 4-fold higher in A549 cells than that of H596 cells (Fig. 6B). Together, these data suggest that the release of EPA in H596 cells from EPA-incorporated PC was much less than in A549 cells due to limited expression and activity of cPLA_2_ in H596 cells, supporting the hypothesis that EPA metabolism is differentially regulated, as suggested by the MALDI-MS analysis.

### EPA altered lipid metabolism in K-ras mouse lung tumor tissues

**Figure 7:**
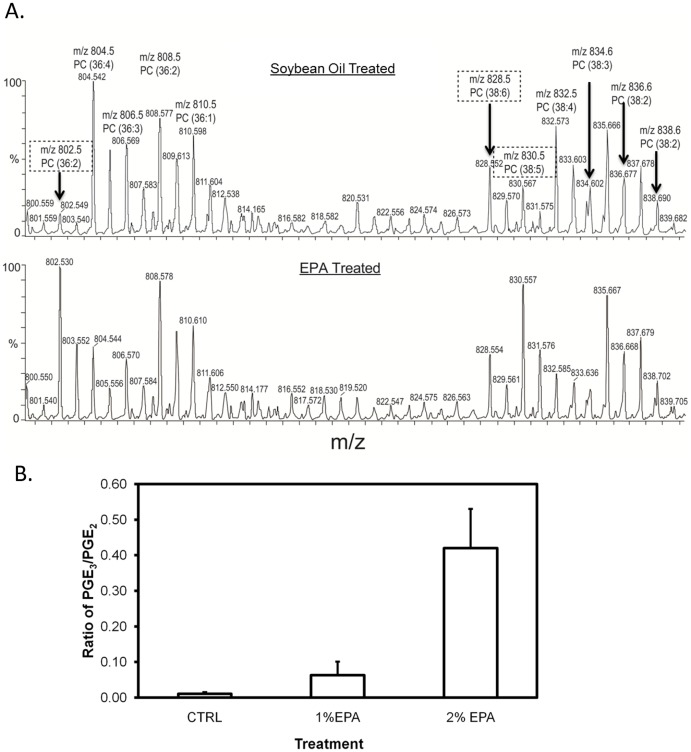
Lipid metabolism in lung tumor tissues from K-ras transgenic mouse. A). MALDI mass spectra collected directly from tumor tissue lysates of K-ras mutant lung tumors, either untreated or treated with EPA. Observed shifts in lipid catabolism from AA to EPA are boxed, showing significant changes in tumor metabolism. B). Ratio of PGE_3_/PGE_2_ extracted from tumor tissue derived from the K-ras transgenic mouse model. PGE_2_ and PGE_3_ were quantified by LC-MS/MS. These data represent the tumor's metabolism shift from producing AA derived PGE_2_ to PGE_3_ from EPA thus eliciting an anti-proliferative effect.

To determine whether MALDI-MS could be used to observe changes in tissue metabolism *in vivo*, after dietary consumption of EPA, experiments were also performed on lung tissue extracts from genetic K-ras mutant mice. The mass spectra collected directly from lung tissue ranging from *m/z* 800−840 are shown in Fig. 7. From the direct tissue analysis with MALDI, the effect of each diet can be observed from the lipid profiles of the tumor. After feeding EPA (Fig. 7A), the corresponding PC species with the EPA (*m/z* 802 and 830) acyl-group was upregulated compared with that in the soybean diet group. Intriguingly, the ratio of PGE_3_ over PGE_2_ was significantly increased from 0.011±0.004 of tissues from soybean-fed mice to 0.063±0.03 (1% EPA) and 0.42±0.11 (2% EPA) of lung tissues derived from mice fed EPA (Fig. 7B). The observation of EPA incorporation into the PC species correlated well with the ratio of PGE_3_/PGE_2_ detected from the same tissue samples by LC-MS/MS, suggesting that EPA led to the tumor's shift in metabolism from producing the proliferative metabolite PGE_2_ to anti-proliferative metabolite PGE_3_ through the COX-2 pathway.

## Discussion

The initial goal of this study was to determine whether the differential response of NSCLC cell lines to EPA treatment could be measured by direct analysis with MALDI-MS. Although the statistical tools employed could not directly discriminate between the two cell lines before treatment, we could successfully differentiate the cell lines based on their mass spectra profiles following EPA treatment. These data have led to an increased understanding of the biological mechanism responsible for the H596 cells' relatively lower sensitivity to EPA treatment than that of A549 cells, even though the expression of COX-2 protein was similar in both cell lines. To our knowledge, this is the first report to directly analyze cancer cells by MALDI-MS, revealing differences in cellular metabolism, which could be an index for EPA-elicited anticancer activities in NSCLC cells. This simple MALDI-MS approach allowed for the interrogation of the lipid signaling pathways operating in NSCLC cell lines.

While using MALDI-MS with no analytical separation has numerous drawbacks, including ion suppression, MALDI matrix-interfering ions, and the inability to separate isobaric endogenous species, the technique still has great potential in its ability for direct measurements from biological samples. This has been exemplified by the recent growth in MALDI imaging MS applications [34]. Further advancements in direct biological analysis by MS techniques, such as matrix-free ionization techniques or atmospheric desorption techniques, will allow for a larger breadth of metabolomic and lipidomic coverage.

These techniques can be especially useful when combining classical quantitative targeted metabolomic approaches. As reported here, using MALDI-MS to investigate differences in lipid metabolism after treating cells with EPA has led to a possible characterization of phenotype of the NSCLC A549 and H596 cells. Additionally, the similar metabolic shift in H596 and A549 cells was also observed in the cells being treated with arachidonic acid, i.e., peak intensity of the PC (36∶4), AA-incorporated PC, was higher in H596 cells than in A549 cells (data not shown). Thus, these results suggest that the differential metabolic patterns generated in the cells were not substrate dependent, rather most likely was dependent on the phenotype of these two cell lines. The MALDI-MS results were substantiated by further interrogation of the COX-2 pathway and its EPA metabolite PGE_3_ using quantitative LC-MS/MS. Given that fatty acids, such as EPA or AA, need to be released from phospholipids in the membrane by cPLA_2_ in order for them to be utilized by COX or lipoxygenases, these data suggest that cPLA_2_ is differentially regulated in these two NSCLC cells. Indeed, we observed both lower expression and activity of cPLA_2_ in H596 than that of A549 cells. We are currently evaluating whether cPLA_2_ is critical for EPA-elicited anti-proliferative activity through its COX-2 metabolite PGE_3_ in NSCLC cells.

The statistical tools used guided us in discriminating between these cell lines and helped us identify a difference in metabolites between treatment groups. PCA and discriminate analysis (data not shown) were used as a preliminary tool to simply identify the differences in the mass spectra of cells or tissues with or without treatment of EPA. We were able to identify some features corresponding to these differences, but direct mass spectral interpretation was eventually utilized. Ultimately, our understanding of the biological implications of PUFA dosing combined with the observed changes in lipid profiles led us to investigate this pathway. While this approach proved useful, it is also cumbersome and challenging due to the complexity of the data, necessity for replicates, and manual interpretation of the results. However, once specific biomarkers (PC lipids in this case) are identified, data processing can become significantly faster and possibly even automated.

The experiments described in this study were performed using an untargeted metabolomics workflow similar to Cho *et al*. [35], in which MS data were collected and statistical tools were used to identify notable features in the data. MALDI-MS data were used without retention time separation to identify significantly changing peaks. MALDI-MS experiments require very little sample (<10 mg), minimal sample preparation, and short analysis time; thus, this technique can be useful in differentiating cell or tissue types, where sample is limited. Indeed, a few studies have been reported using MALDI for such tissue types, including identifying primary cancer sites from analysis of metastatic lesions, cancer stage determination from gliomas, and intrasurgical normal/cancer tissue discrimination [18], [21]. Using these advanced MS approaches will lead the way in small molecule biomarker discovery and could potentially be used to determine treatment effectiveness at a very early stage.

A better understanding of the role of the COX pathway in carcinogenesis could lead to the development of more precise therapies for cancer prevention and treatment. Higher levels of PGE_2_ have been shown to significantly increase cell proliferation, invasion, and metastasis in various cancers, including lung cancer. Conversely, increasing PGE_3_ concentrations has been shown to correlate well with inhibition of cell proliferation in cancer cells, as the COX pathway is shifted from the production of PGE_2_ to that of PGE_3_ from the substitution of EPA for AA [22], [36]. This notion was supported by previously published results from our laboratory as well as the IC_50_ plot shown in Fig. 2. To our surprise, even though both A549 and H596 cells expressed similar levels of COX-2 enzymes, the response of H596 to EPA was much less sensitive than that of A549 cells. Identifying the biological mechanism as to why the H596 cells were less sensitive to EPA treatment than the A549 cells can enhance our understanding of the precise molecular mechanism(s) associated with the EPA-elicited biological effect. Our data clearly suggest that other factors, such as cPLA_2_, that regulate the release of substrate for biosynthesis of PGE_3_ could be equally important as the COX-2 enzymes. These data also suggest significant overlap in responses observed when comparing *in vitro* models compared with *in vivo* models. When mice were treated with EPA diet, similar metabolic shifts were observed in the tumor as was observed in the cellular experiments. Using MALDI-MS provides a snapshot of the tumor's metabolism (particularly lipid metabolism), which allows us to estimate the amount of EPA incorporating into the tumor environment and ultimately could help predict the effectiveness of these particular omega-3 fatty acids in NSCLC treatment. We are currently evaluating the antitumor efficacy of EPA in both H596 and A549 xenograft models to further delineate the role of COX-2 metabolite, PGE_3_ in NSCLC.

In conclusion, characterizing transformed cells or neoplastic tissues should not be solely based on genomic or proteomic approaches. In many instances, these data cannot provide a snapshot into the specific metabolism of a particular cell type, especially when cell metabolism can be rapidly shifted by a given treatment stimulus. Furthermore, it is widely regarded that changes in the metabolite profile in direct relation to biological activity offers the most accurate link to phenotype changes. This study exemplifies the importance of investigating cancer on both a lipidomic and a metabolomic level and the potential to combine these results with genomic and proteomic data, particularly in efforts to delivery personalized cancer therapy.

## Supporting Information

Figure S1 Mass spectra collected directly from each cell line, untreated A549 (A) and H596 (B) versus 50 µM EPA-treated A549 (C) and H596 (D). Consistent with the PCA data analysis, differentiation between A549 and H596 is difficult to observe before treatment with EPA. Numerous new *m/z* peaks are observed in the H596 cell line after treatment with EPA.
(PPTX)

Figure S2 MS/MS spectrum of *m/z* 802.5 collected from the Waters QTOF.
(PPTX)
