# Supplementary figures and images for: Changes in Cancer Cell Metabolism Revealed by Direct Sample Analysis with MALDI Mass Spectrometry
Source: PLoS One. 2013 Apr 26;8(4):e61379. doi: 10.1371/journal.pone.0061379 (PMC3637300; doi:10.1371/journal.pone.0061379)

## Slide 1
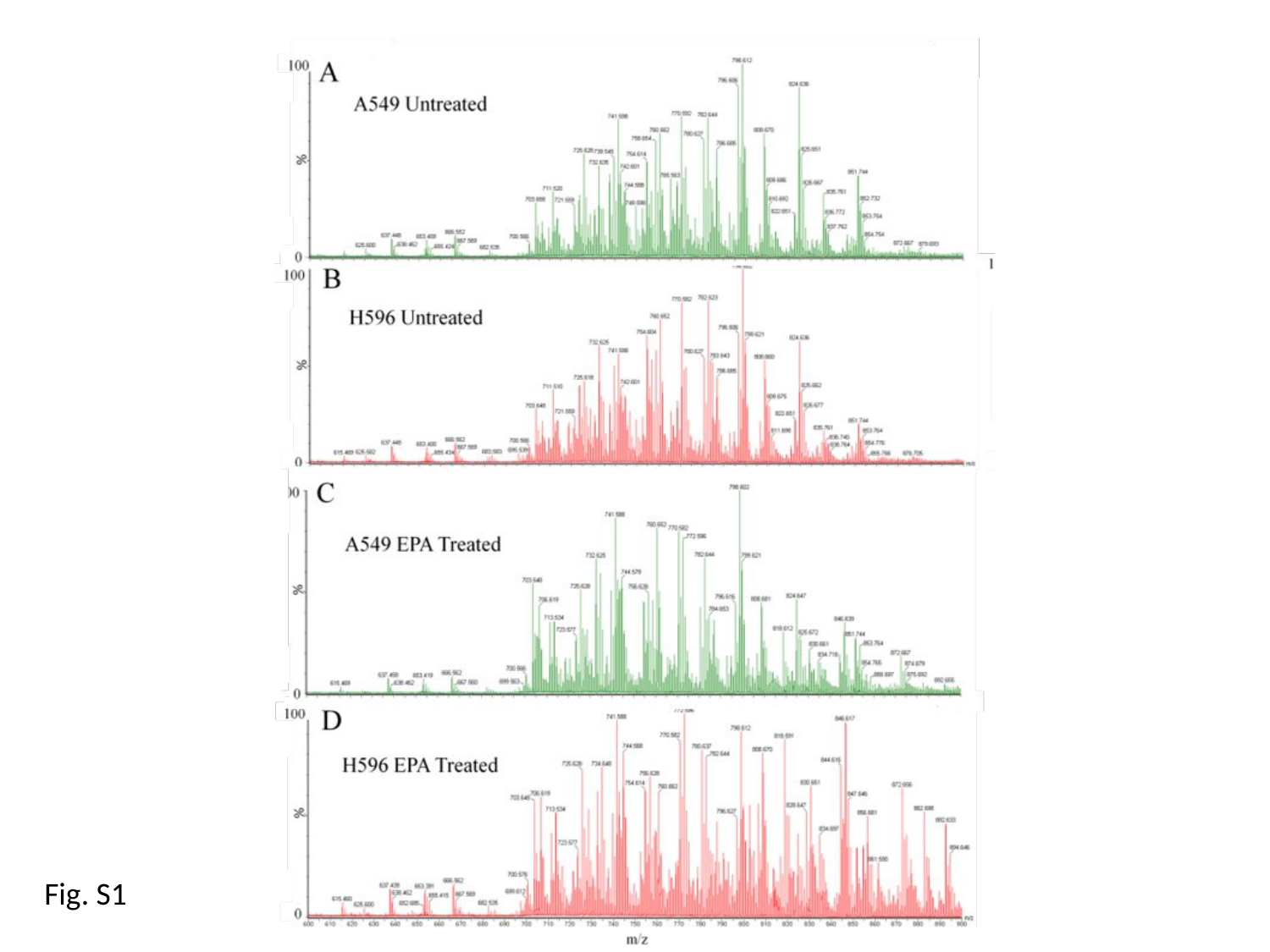

Fig. S1

Supplement: Figure S1 — Mass spectra collected directly from each cell line, untreated A549 (A) and H596 (B) versus 50 µM EPA-treated A549 (C) and H596 (D). Consistent with the PCA data analysis, differentiation between A549 and H596 is difficult to observe before treatment with EPA. Numerous new m/z peaks are observed in the H596 cell line after treatment with EPA. (PPTX) [file pone.0061379.s001.pptx]

## Slide 1
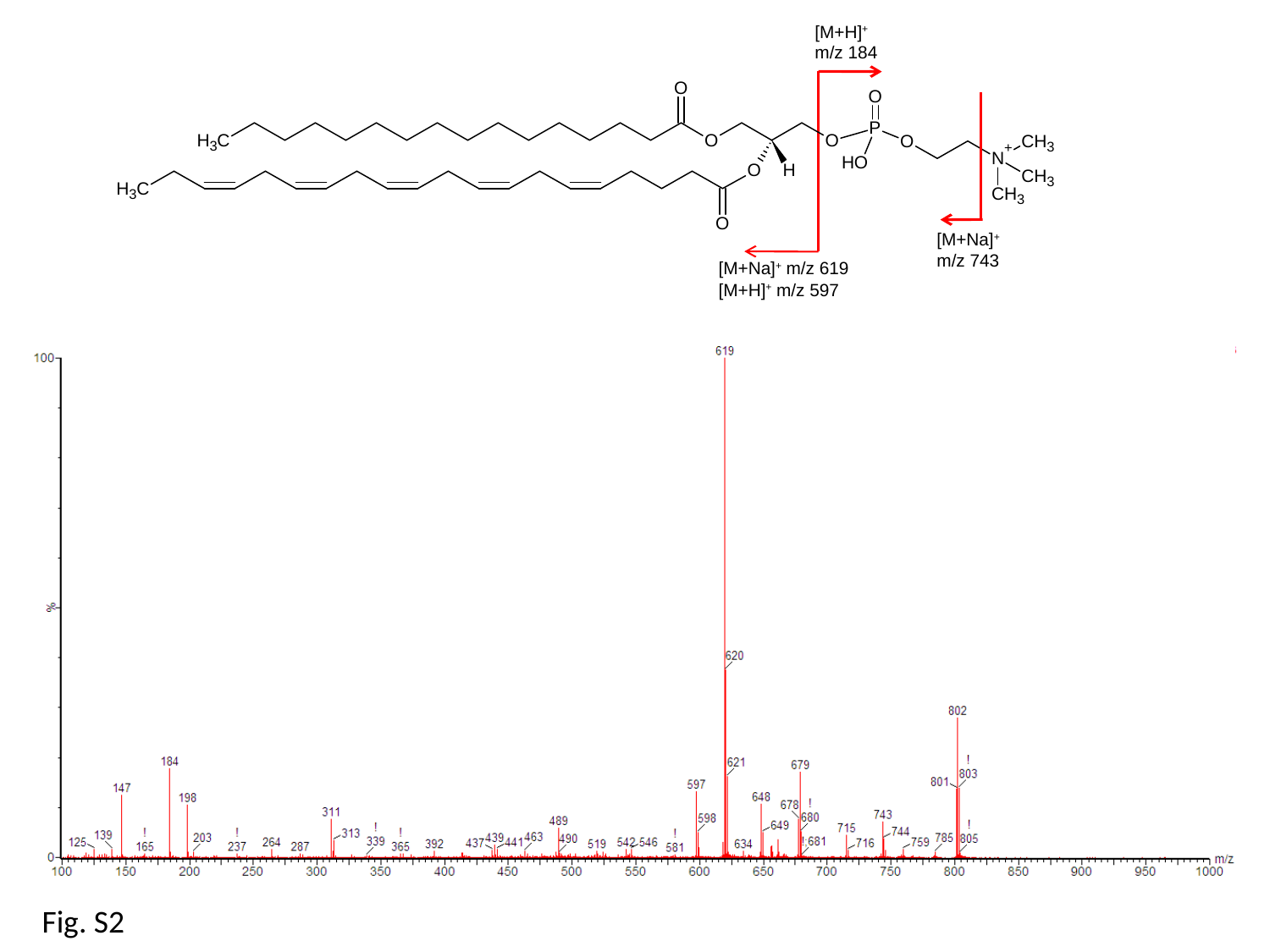

[M+H]+
m/z 184
[M+Na]+
m/z 743
[M+Na]+ m/z 619
[M+H]+ m/z 597
Fig. S2

Supplement: Figure S2 — MS/MS spectrum of m/z 802.5 collected from the Waters QTOF. (PPTX) [file pone.0061379.s002.pptx]
